# 3D-Printed Veterinary Dosage Forms—A Comparative Study of Three Semi-Solid Extrusion 3D Printers

**DOI:** 10.3390/pharmaceutics12121239

**Published:** 2020-12-19

**Authors:** Erica Sjöholm, Rathna Mathiyalagan, Dhayakumar Rajan Prakash, Lisa Lindfors, Qingbo Wang, Xiaoju Wang, Samuli Ojala, Niklas Sandler

**Affiliations:** 1 Pharmaceutical Sciences Laboratory, Faculty of Science and Engineering, Åbo Akademi University, Tykistökatu 6A, 20520 Turku, Finland; rathna.mathiyalagan@abo.fi (R.M.); dhayakumar.rajanprakash@abo.fi (D.R.P.); lisa.lindfors@abo.fi (L.L.); xiaoju.wang@abo.fi (X.W.); niklas.sandler@nanoform.com (N.S.); 2 Johan Gadolin Process Chemistry Centre, Åbo Akademi University, Piispankatu 8, 20500 Turku, Finland; qingbo.wang@abo.fi; 3 Oulun Keskus Apteekki, Isokatu 45, 90100 Oulu, Finland; samuli.ojala@saunalahti.fi; 4 Nanoform Finland Oyj, Viikinkaari 4, 00790 Helsinki, Finland

**Keywords:** 3D printing, semi-solid extrusion 3D printing, additive manufacturing, extemporaneous manufacturing, drug delivery, personalized dosage forms, veterinary medicine, prednisolone

## Abstract

Currently, the number of approved veterinary medicines are limited, and human medications are used off-label. These approved human medications are of too high potencies for a cat or a small dog breed. Therefore, there is a dire demand for smaller doses of veterinary medicines. This study aims to investigate the use of three semi-solid extrusion 3D printers in a pharmacy or animal clinic setting for the extemporaneous manufacturing of prednisolone containing orodispersible films for veterinary use. Orodispersible films with adequate content uniformity and acceptance values as defined by the European Pharmacopoeia were produced with one of the studied printers, namely the Allevi 2 bioprinter. Smooth and flexible films with high mechanical strength, neutral pH, and low moisture content were produced with a high correlation between the prepared design and the obtained drug amount, indicating that the Allevi 2 printer could successfully be used to extemporaneously manufacture personalized doses for animals at the point-of-care.

## 1. Introduction

Veterinary drug treatment is often challenging due to size and body mass variations, as well as pharmacokinetic inter-species variability. The availability of approved veterinary products is relatively small, consequently leading to the need for extemporaneous manufacturing. There are several ways to define the practice of the extemporaneous preparation of medicines. In this article, the term extemporaneous manufacturing will be used, but it may also be referred to as extemporaneous preparation, extemporaneous compounding, or simply compounding. Generally, extemporaneous manufacturing is necessary when a therapy cannot be provided by commercially available products to a specific patient. By extemporaneous manufacturing, dosage forms that may be easier for a pet to tolerate and for an owner to administer can be provided, thus encouraging compliance. Mixing two or more approved drug products together into a single dosage form, changing the dosage form of an approved drug product, adding patient-preferred flavoring to an approved drug product, or preparing a dosage from bulk ingredients are all considered extemporaneous manufacturing [1]. European Pharmacopoeia defines extemporaneous preparations as pharmaceutical preparations that are individually prepared for a specific patient or patient group and supplied to the patient after preparation. In contrast, stock preparations are prepared in bulk in advance and stored until needed [2]. Extemporaneous manufacturing generally takes place at pharmacies in accordance with the prescription provided by a licensed veterinarian.

There are benefits when it comes to the extemporaneous manufacturing of veterinary drug therapies, but there are also risks. These risks are due to preparation errors, contamination, chemical and physical stability, and a lack of bioavailability in the target patient. As an example, The Missouri Board of Pharmacy annually tests and reports extemporaneously manufactured drug preparations from Missouri licensed pharmacies. In Table 1, the results obtained between the years 2006–2019 are gathered. The Missouri Board of Pharmacy has considered an acceptable potency range of +/−10% of the expected potency unless a USP monograph states a different range for specific preparations. The results indicate that every fifth extemporaneously manufactured dosage form does not meet the targeted strength criteria. Analyzed potencies ranged from not containing any active ingredient at all (years 2006 and 2009) up to a potency of 450.4% (the year 2007) [3]. To assure the quality of the produced drug doses, quality assurance should be implemented for extemporaneously manufactured doses.

A challenge when extemporaneously manufacturing veterinary medication is finding a suitable dosage form. Flavoring plays a crucial role in successful administration, as well as the selection of an appropriate drug delivery system. In the present work, the prepared dosage forms are intended for cats and dogs, and therefore liver powder was chosen as the flavoring agent. Other flavors preferred by cats and dogs are bacon, beef, cheese, cod liver oil, marshmallow, molasses, and peanut butter [4].

Prednisolone is a glucocorticoid used in the treatment of arthritis, asthma, skin disorders, allergic dermatoses, and other inflammatory conditions in both cats and dogs. Dogs are dosed with 0.5–1 mg/kg/day prednisolone. However, there is evidence that cats have fewer glucocorticoid receptors and express a higher resistance to glucocorticoid treatment [5]; therefore, cats require larger doses compared to dogs and are treated with doses of 1–2 mg/kg/day. At present, prednisolone tablets can only be found in the strengths of 5, 20, and 40 mg in Finland.

There is a dire demand for smaller doses of prednisolone to treat cats and small dog breeds. Currently, pharmacies are extemporaneously manufacturing capsules manually to answer this unmet need. Manufacturing capsules is labor extensive, and a novel manufacturing technique is desired. For the production of personalized doses at the point-of-care, the manufacturing technique has to be precise, rapid, and flexible. Furthermore, it should be simple, robust, and of low-cost [6]. Lately, printing technologies have emerged in the pharmaceutical field as manufacturing methods for the production of personalized doses on-demand [7]. Three-dimensional (3D) printing or additive manufacturing is the production of a 3D object by the layer-by-layer approach. There are many different 3D printing technologies, and several of them have been investigated for pharmaceutical manufacturing. Fused deposition modeling (FDM) 3D printing prints the desired 3D object by melting a pre-made drug-loaded filament, and by changing the infill [8,9] or the geometry [10], oral tablets with different release kinetics have been produced. Tablets with linear release profiles [11] and fast disintegrating tablets [12] have been investigated by utilizing powder bed fusion 3D printing. Semi-solid extrusion (SSE) 3D printing, the method used in this study, has been explored for the production of polypills [13], controlled-release bi-layered tablets [14], orodispersible films [15,16], solid self-microemulsifying drug delivery systems (SSMEDDS) [17], and suppositories [18].

Printing technologies have been used in veterinary medicine by the production of animal prosthetics, medical implants, and biological tissue replacement [19]; veterinary drug dosage forms have, to the best of our knowledge, not yet been produced. Prednisolone has previously been printed by thermal inkjet printing [20], piezoelectric inkjet printing [21], and FDM 3D printing [22,23]. These techniques may face difficulties in the production of prednisolone-containing drug doses for veterinary use. Inkjet printing is known to produce low drug doses, and it can be challenging to achieve high enough levels of prednisolone to reach therapeutic doses. FDM 3D printing uses high temperatures, which may cause problems for a heat-sensitive compound like prednisolone. SSE is an extrusion-based technique like FDM, but instead of melting a solid filament, a semi-solid material like a gel or a paste is extruded at low temperatures. The benefit of SSE compared to FDM is that there is no need to operate at highly elevated temperatures, which is unsuitable for thermolabile compounds, and high levels of drug-loaded dosage forms can be produced. FDM-printed 3D objects solidify upon cooling, while SSE-printed objects require a drying or a cross-linking step [24,25].

This study aims to explore three different SSE 3D printers to provide a more automated approach for extemporaneously manufacturing personalized orodispersible films (ODFs) for veterinary use utilizing SSE 3D printing.

## 2. Materials

Prednisolone (PRE) (Sigma-Aldrich, 99%, Shanghai, China) was used as the active pharmaceutical ingredient in this study. Pure liver powder (LP) (CC Moore & Co Ltd., Stalbridge, UK) was added as a taste-enhancing agent to render the dosage form more appealing to animals. To enhance the viscosity of the printing ink, three polymers were investigated, namely polyethylene oxide (PEO) (Sigma Aldrich, St. Louis, MO, USA) and two grades of hydroxypropyl cellulose (HPC) (Klucel™ EXF and LF, molecular weight 80,000 and 95,000, respectively) which were kindly provided by Ashland (Rotterdam, The Netherlands). Ethanol (EtOH) (Etax A 94 w-%, Altia Oyj, Rajamäki, Finland) was used in combination with purified water (MQ) (Milli-Q water, Turku, Finland) as a solvent for the formulations. For the preparation of buffer solutions and simulated saliva, the following chemicals were purchased: potassium dihydrogen phosphate from Merck KGaA (Darmstadt, Germany), disodium hydrogen phosphate from Honeywell Fluka (Seelze, Germany), 37% hydrochloric acid from Fischer Scientific UK (Loughborough, UK), and sodium chloride from Sigma Aldrich (St. Louis, MO, USA. For the preparation of artificial skin, the following chemicals were used: gelatin from porcine skin (gel strength ~175 g Bloom, Type A) and glycerol was purchased from Sigma Aldrich (St. Louis, MO, USA), Natrosol™ 250M PHARM and ProLipid™ 141 was kindly provided by Ashland (Schaffhausen, Switzerland), 37% formaldehyde solution was obtained from J.T. Baker (Deventer, The Netherlands), and sodium hydroxide was purchased from VWR International BVBA (Leuven, Belgium). All water used in this study was purified by a Millipore SA-67120 system from Millipore (Molsheim, France), and all reagents were of analytical grade and used without further purification.

## 3. Methods

### 3.1. Preparation of Orodispersible Films

The innovative manufacturing method, semi-solid extrusion (SSE) 3D printing, was investigated and evaluated for the purpose of extemporaneously manufacturing orodispersible films (ODFs) for veterinary treatment at the point-of-care. Three different SSE 3D printers were studied and compared, namely a Biobot 1 3D bioprinter (Allevi, Philadelphia, PA, USA) equipped with a compressor operating through air pressure, a Bocusini 3D food printer (Procusini, Freising, Germany) and a Zmorph multitool 3D printer (Zmorph, Wrocław, Poland), both operating through the addition of mechanical pressure to a plunger. The Biobot 1 and the Bocusini printers have changed their names to Allevi 1 and Procusini, respectively. Throughout the text, we will be referring to the printers by their old names. Utilizing SSE, a pharmaceutical ink is first produced. This ink is then printed according to a pre-determined design utilizing pressure, and upon solidification, a drug dosage form is obtained.

#### 3.1.1. Ink formulation

Several ink solutions with different polymers and combinations were studied to prepare a working ink formulation for SSE 3D printing of veterinary medication with immediate release utilizing the three different printers. Polyethylene oxide (PEO) and two grades of hydroxypropyl cellulose (HPC) were studied in combination with various percentages of liver powder (LP) and prednisolone (PRE). The ratio between purified water and ethanol (MQ:EtOH) in the solvent was also investigated and evaluated. Desired attributes of a good ink are easy preparation (preferably no heating required), a smooth and even texture without lumps, easy to pour, and not too sticky. The ink must keep the pre-determined shape after extrusion and not spread out. The polymer acts as a film-forming agent and gives the film flexibility. It makes up most of the dry weight of the film. The three tested polymers are common in mucoadhesive and/or orodispersible films and have good film-forming properties [26]. A film-forming solution suitable for all three printers was chosen in order to identify differences between the printers.

In conclusion, for the initial print, a formulation consisting of 1% of the active pharmaceutical ingredient (API) prednisolone, 1% of the taste-enhancing agent LP, and 24% of the film-forming agent HPC EXF was chosen, this formulation is referred to as the drug-loaded (DL) solution with LP. As reference, a placebo solution with 24% HPC and 1% LP was prepared called the unloaded (UL) solution with LP. All ingredients were dissolved in a 1:1 MQ:EtOH (*v/v*) solvent mixture on a magnetic stirrer for 24 h at room temperature. When homogenous solutions were obtained, the solutions were placed in 10 mL disposable syringes (BD Plastipak TM Luer-Lok, Becton Dickinson S.A., Madrid, Spain) to be printed with the Biobot printer and 50 mL disposable syringes (Henke-Ject Luer-Lok, Henke sass wolf GmbH, Tuttlingen, Germany) to be printed with the Bocusini and the Zmorph printers. Tips (1.5-inch 20 G) were attached (Quantx precision dispense tips, Fisnar, Germantown, WI, USA) to the syringes. The solution-filled syringes were left to stand at room temperature until all air bubbles could be removed prior to printing.

For the second print, the effect of the LP was investigated, and four new solutions were prepared. Two of the solutions were without LP and instead had a higher HPC concentration, namely the solutions referred to as the DL solution consisting of 25% HPC and 1% PRE and the UL solution consisting of only 25% HPC. The other two solutions consisted of the higher HPC amount in combination with LP. These two solutions are referred to as the DL nonprintable solution with LP (1% PRE, 25% HPC, and 1% LP) and the UL nonprintable solution with LP (25% HPC and 1% LP). The content of all the prepared solutions can be seen in Table 2. As a comparison, a solution containing 2% PRE (2% DL solution) is also prepared.

#### 3.1.2. Rheology

Rheology measurements were performed on UL and DL solutions with and without LP, as well as on the UL and DL nonprintable solutions. The measurements were conducted by a MCR 702 MultiDrive rheometer (Anton Paar GmbH, Ashland, VA, USA) with a PP25 parallel plate (diameter and measuring gap of 25 mm and 0.5 mm, respectively) at 23 °C. Viscosity curves were obtained through shear flow measurement of shear rate with a logarithmic ramp of 0.01–1000 s^−1^, with 1 s per data point. The samples were pre-sheared at 1 s^−1^ for 20 s and equilibrated for 60 s before the measurement. The thixotropic behavior of the formulations was analyzed by shearing the sample at 1 s^−1^ for 60 s, followed by shear at 700 s^−1^ for 10 s, and then at 1 s^−1^ for 120 s. The samples were pre-sheared at 10 s^−1^ for 60 s and equilibrated for 60 s before measurement.

#### 3.1.3. Film Design

For the first part of the study, films of one size were printed with each printer to compare content uniformity. The same solution was used for all printers, but for each printer, an optimized design was used. The design used for the printers was chosen by changing the design for each printer until the same wet weight of the printed films was achieved for the same solution. For Biobot printing, six squares with dimensions 20 × 10 × 2 mm were designed using a computer-aided design software (Fusion 360, version 2020, Autodesk, San Rafael, CA, USA). The design was saved as a .stl file and imported into the slicer software (RepertierHost v1.6.1, Hot-World GmbH & Co. KG, Willich, Germany), where the print settings were set, and the g-code was generated. When printing with the Bocusini food printer, a design of six squares with dimensions 20 × 10 × 1 mm was designed in Fusion 360. The .stl file was imported to Procusini Club under “Pasta” so no heat was added, and the g-code was generated. For the Zmorph printer, the paste extruder can only read 2D designs, and therefore six squares with dimensions 20 × 10 mm with 55 vertical lines were designed in Fusion 360. The design was saved as a .dmx file, imported into the slicer software (Voxilizer 2.0.0, Wrocław, Poland), and the g-code was generated after the print settings were set.

For the second part of the study, five different doses were printed by altering the *Y*-axis of the design. The film sizes were decided based on the assumption of what is handleable and on the assumed preferences of the animals. The sizes of the five designs were the following: 10 × 15 × 1 mm, 15 × 15 × 1 mm, 20 × 15 × 1 mm, 25 × 15 × 1 mm, and 30 × 15 × 1 mm, which will be referred to as films with size 10, 15, 20, 25, and 30, respectively. For the puncture, disintegration, and tensile tests, the following designs were prepared and printed, respectively: 20 × 20 × 1 mm, 30 × 40 × 1 mm, and 100 × 10 × 1 mm. All designs were made using the computer-aided design software Fusion 360.

#### 3.1.4. Semi-Solid Extrusion 3D Printing

SSE 3D printing was performed with three different printers. The Biobot and Bocusini printers were both utilized as received, but the Zmorph printer was modified to replace the original 100 mL syringe with a 50 mL luer lock syringe. The original syringe is attached to the printer by FDM 3D printed parts, including the plunger. The smaller syringe was attached to the printer by designing new parts to fit the dimensions of the smaller syringe, as well as a new plunger. The designs were made in Fusion 360 and printed out with the Zmorph printer that has the capability to print both FDM and SSE 3D prints. The stepper motor in the Zmorph printer was exchanged for a Nema 23 (Stepperonline, Nanjing, China) to obtain higher torque.

The solutions were placed in 10- and 50-mL disposable syringes attached with 20 G dispensing precision tips and printed on pieces of transparency sheets (Clear transparent X-10.0, Folex, Köln, Germany). Prednisolone-containing films were printed with the Biobot printer with the following settings: 2 layers were printed with a layer height of 1 mm, one vertical shell and filled using a rectilinear fill pattern with a 45° fill angle. The infill density was set to 100%, and the infill overlap to 100%. The printing was performed at 96 PSI and with a print speed of 5 mm/s. For the Bocusini food printer, it is not possible to change any settings other than the choice of material, which then sets the temperature. We chose to print without heat, which for this printer was the “pasta” setting. The design was printed in one layer. The Zmorph printer was set to print with 100% thickness, 1-layer count, 2 mm layer height, 2 mm path width, the retraction was set to off, and the print speed was set to 10 mm/s.

These three printers were compared regarding printability, speed, and user-friendliness, but primarily, the obtained content uniformity between the printers was compared to determine which printer would work the best in a pharmacy or an animal clinic setting. Six squares were printed at a time, equaling half a batch. The printing was performed twice, providing one batch before the printers were restarted, and two new sets of six squares were printed two more times, producing batch two and three. The printing was performed in this manner to study the content uniformity within a batch but also between the batches. The time it took to print six squares with each printer was recorded and compared. The printed films were then left to dry for two days under ambient conditions before drug content was determined and content uniformity was calculated. After obtaining the results, one printer was chosen for further studies.

From the uniformity of the content results, the Biobot 1 SSE 3D printer was chosen for further studies. For the second print, the print time was decreased by replacing the tip with a shorter one (0.25-inch 21 G tip Quantx precision dispense tips, Fisnar Europe, South Lanarkshire, Scotland, UK) to increase the flow. The designs were then printed in one layer instead of two, with a pressure of 84 PSI and a print speed of 10 mm/s.

### 3.2. Drug Content

The drug content of the printed films was determined to evaluate the amount of drug obtained by utilizing the different printers. Drug content was spectrophotometrically determined by placing one accurately weighed ODF in 100 mL of purified water and shaken (Multi-shaker PSU 20, Biosan, Riga, Latvia) at 150 rpm for a minimum of 3 h (average ± standard deviation (SD), *n* = 10) before measuring the absorbance with UV-Vis (UV-6300PC Double Beam Spectrophotometer, VWR International BVBA, Leuven, Belgium) at 246 nm. A calibration curve was obtained by taking different aliquots of a 100 µg/mL prednisolone stock solution, and the absorbance was subsequently spectrophotometrically analyzed at 246 nm against placebo as the baseline. All twelve samples per batch for each printer was analyzed. The uniformity of content (UC) of single-dose preparation was calculated according to the European Pharmacopeia (European Pharmacopoeia 10.0) 2.9.6, test B [27], and the acceptance values (AV) were calculated as described in European Pharmacopoeia 2.9.40 [28]. Utilizing these values, the obtained drug amounts of the different printers were compared, and the batch-to-batch reliability was evaluated. The UC test complies with the requirements if no more than one individual content is outside 85–115% of the average content, and all individual contents are between 75–125%. An additional 20 units should be tested if two or three individual dosage units are outside the 85–115% range of average content. The test fails to comply with the requirements if more than three individual contents are outside 85–115% of the average content. The AV was calculated using the acceptability constant k = 2.4 and T = 100%. To meet the requirements and pass the test, the AV (L1) should be ≤15.0. In the present study, the UC and AV were based on six replicates per half batch, adding up to twelve replicates per batch per printer. An additional 20 dosage forms were not analyzed.

### 3.3. Weight, Thickness, and Appearance of the Dosage Forms

The thickness of the films was measured at five locations (all corners and the middle of the film) utilizing a caliper (CD-6 “CX, Mitutoyo, Kawasaki, Japan), and the weight of the films was determined using an analytical balance (AND GH-252, A & D Instruments Ltd., Tokyo, Japan). Average and standard deviations were calculated and compared within the batch, between the batches, and between the printers. The appearance of the prepared ODFs printed with the three different printers was visually evaluated and photographed. Regular pictures were taken with a mobile phone, and microscopy pictures of the surface were taken with a handheld 5 MP digital microscope (Bodelin technologies, Oregon, OR, USA) with the software MicroCapture pro version 2.2 (Oregon City, OR, USA). The surface morphology of the prepared films was studied with a scanning electron microscope (SEM) (LEO Gemini 1530, Carl Zeiss AG, Oberkochen, Germany) equipped with a Thermo Scientific UltraDry Silicon Drift Detector. All samples were coated with carbon by a vacuum evaporator prior to scanning. SEM images were attained at an accelerated voltage of 8 kV with 50× magnification. The images were taken using the secondary electron detector, and the pressure was kept at 2 × 10^−5^ mbar during scanning.

### 3.4. Mechanical Testing

Three different tests were conducted to investigate the mechanical properties of the films, namely: puncture test tensile strength, and folding endurance. The mechanical properties of ODFs affect the performance, and ODFs should possess sufficient mechanical properties to endure handling and transport.

#### 3.4.1. Puncture Test

The film burst strength and the flexibility of the films were determined with a puncture test. UL and DL films with and without LP that were 20 × 20 × 1 mm in size were measured with the Texture Analyser TA-XTplus (Stable Micro Systems, Godalming, UK) equipped with a 10 kg load cell, set up with the film support rig (Stable Micro Systems, Surrey, UK), and a 5 mm stainless steel ball probe (spherical probe SMS P/5S, TA.XT.Plus Texture Analyser, Surrey, UK). The test was performed by the apparatus bringing the upper probe down with a travel speed of 2 mm/s until the probe touched the sample, and the trigger force of 0.049 N was achieved. When reaching the trigger force, the probe continued to move down with a constant speed of 1 mm/s until a distance of 10 mm was reached. As result, the film burst strength (in N) and the distance to burst (in mm), which can be interpreted as the flexibility, were gained. Data collection and calculation were performed using Stable Micro Systems software (2013 version 6.1.4.0, TA.XT.Plus Texture Analyser, Surrey, UK). The measurements were performed six times under ambient conditions (23.4 ± 0.3 °C and 38.0 ± 2.1 relative humidity (RH)%); average and standard deviations were calculated.

#### 3.4.2. Tensile Strength

A tensile test was performed on UL and DL films that were 100 × 10 × 1 mm in size with and without LP using the TA.XT.Plus Texture Analyser to measure the films’ resistance to longitudinal pulling. The film was fixed between two self-tightening roller grips (Stable Micro Systems, Surrey, UK), so the exposing area of the film was set to 10 × 80 mm, the lower clamp was held stationary, and the film was pulled apart by the upper clamp with a speed of 0.10 mm/s to a distance of 60 mm and with a trigger load of 0.029 N. Maximum tensile force and the distance of pulling at the maximum force point was recorded. Data collection and calculation were performed using Stable Micro Systems software. The measurements were performed six times under ambient conditions (23.6 ± 0.1 °C and 35.0 ± 0.2 RH%); average and standard deviations were calculated. The percentage of elongation was calculated from the elongation amount compared to the original sample length of 80 mm.

#### 3.4.3. Folding Endurance

The value of folding endurance was obtained by folding the film at the same place until breaking or cracking. The folding endurance test in this study was performed manually by repeatedly folding the 20 × 20 × 1 mm films at a 180-degree angle at the same place until the film cracked or broke. The test was performed in triplicate under ambient conditions (24.3 ± 0.1 °C and 28.2 ± 1.8 RH%); average and standard deviations were calculated.

### 3.5. Moisture Content

There are not yet any pharmacopeial specifications for the preferred moisture content of ODFs [29,30]. Some residual moisture is desirable in ODFs; the presence of water gives flexibility to the films, as completely dry films are inclined to be brittle and have reduced handleability. However, too high-water content may yield sticky films and increase the risk of microbial growth [30]. It is challenging to compare and determine the optimal moisture content of ODFs. With different drug substances, film-forming polymers and other excipients have different properties. To determine the moisture content of the prepared dosage forms, the loss of drying was investigated by a moisture analyzer (Radwag Mac 50/NH, Radom Poland). Samples of approximately 0.5 g were placed on an aluminum pan and heated up to 120 °C. The endpoint of the test was set to when the change of mass was less than 1 mg/min, and the measurement had reached equilibrium. The mass-% weight loss corresponding to moisture evaporation was recorded, the measurements were performed under ambient conditions (23.9 ± 0.1 °C and 34.6 ± 0.3 RH%), and the average and standard deviations were calculated (*n* = 3).

### 3.6. Surface pH

For a comfortable mouthfeel, the surface pH of the orodispersible films should be close to neutral. In this study, the surface pH was performed at room temperature by adding 1 mL of purified water on top of unloaded and drug-loaded films, with and without LP, and letting it sit for 30 s before measurement. The pH was noted after bringing the electrode of the pH meter (Mettler Toledo FE20, Mettler Toledo AG, Zurich, Switzerland) in contact with the solution and allowing equilibration for 1 min. The average and standard deviations of the three measurements was calculated.

### 3.7. Adhesiveness Analysis

In vitro mucoadhesive strength was measured by measuring the required force to detach a 10 × 10 × 1 mm film from artificial skin wetted with artificial saliva using the TA.XT.Plus Texture Analyser. Artificial skin was produced in accordance with the procedure described by Lir et al. [31]. The mixture was cast on a transparency sheet and dried under ambient conditions for 48 h. Simulated artificial saliva with a pH of 6.8 consisted of NaCl (8 g/L), KH_2_PO_4_ (0.19 g/L), and Na_2_HPO_4_ (2.38 g/L). The test was performed in accordance with Tejada et al. [32] with slight modifications. A 20 × 20 mm artificial skin piece was fixed to the film support rig, and a 10 × 10 mm film sample was attached by double-sided tape to the upper cylindrical probe (10 mm Delrin cylinder probe, SMS P/10, Stable Micro Systems, Surrey, UK). The test was performed by first hydrating the artificial skin by placing 0.1 mL artificial saliva onto the artificial skin fixed in the film support rig for 10 min. The upper cylindrical probe was then brought down until the orodispersible film was in contact with the artificial skin. After 5 min of film contact, the measurement started by moving the upper probe upwards with a speed of 0.10 mm/s, pulling the orodispersible film from the artificial skin, and measuring the pulling strength needed to detach them. Adhesiveness strength, work of adhesion, and travel distance were recorded by the Stable Micro Systems software under ambient conditions (23.0 ± 0.2 °C and 33.7 ± 1.1 RH%), and average and standard deviations were calculated (*n* = 6).

### 3.8. Disintegration

**Figure 1:**
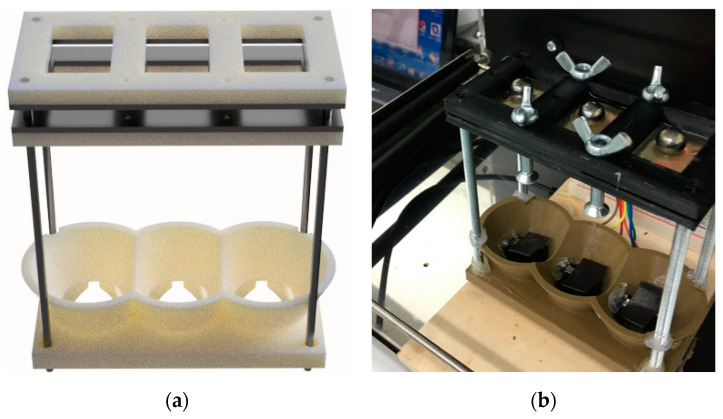
An in-house made slide frame and ball disintegration apparatus. (**a**) Design of the disintegration apparatus and (**b**) the disintegration test setup.

To determine the disintegration of the ODFs, a custom-made apparatus based on the slide frame and ball measurement device from Steiner et al. [33] was designed and built. Two frames with three 20 × 30 mm holes and a bottom basket were designed with Fusion 360 and printed with the Zmorph FDM 3D printer. The parts were attached together with 3 mm linear screws (Figure 1a). The apparatus was placed in a shaking incubator (Unitron plus Incubator shaker, INFORS AG, Bottmingen, Switzerland) set to 37 °C and 50 rpm. The test was conducted by placing a set of three 30 × 40 × 1 films between the frames of the device and adding 100 µL of purified water on each film before placing a 10 mm bearing steel ball (Kento OY, Kokkola, Finland) in the middle of each film, and adding 3 mL of purified water (Figure 1b). The time was recorded until the ball broke the film and hit the switch that turned off the timer. Disintegration time was determined for UL and DL films with and without LP. Average and standard deviations were calculated (*n* = 6).

### 3.9. In Vitro Dissolution

The European Pharmacopoeia recommends using one of the tests found in the general chapter “2.9.3. Dissolution of solid dosage forms” for the dissolution testing of ODFs; therefore, the test used for conventional-release solid dosage forms with apparatus 2 (paddle apparatus) was utilized [34]. In vitro drug release studies were conducted on pure substance prednisolone (as received) and on the UL and DL films with and without LP to study the drug release behavior of the dosage form. The weight and thicknesses of the films were documented prior to the dissolution study. The films were placed in vessels containing 500 mL media, which were immersed into a dissolution bath (Sotax AT 7 smart, Basel, Switzerland) set to 37 °C and equipped with paddles rotating with a speed of 100 rpm. At predefined time-points, aliquots of release media were automatically withdrawn with the use of a pump (Sotax CY 6, Basel, Switzerland). The automatically withdrawn samples were filtered (glass microfiber filter GF/B, GE Healthcare Life Sciences, Cheshire, UK) prior to absorbance measurement at 247 nm utilizing an online UV-Vis spectrophotometer (Lambda 35, PerkinElmer, Singapore). The average cumulative percent drug release (*n* = 3) was calculated based on the results obtained from the content measurements. The drug release kinetics were determined by applying mathematical models to the drug release process. The obtained drug release was plotted according to zero order (cumulative % drug released vs. time), first order (log cumulative % drug remaining vs. time), Higuchi model (cumulative % drug released vs. square root time), Hixson–Crowell model (W_o_-W_t_ vs. time), and Korsmeyer–Peppas model (log cumulative % drug released vs. log time) equations [35]. The release exponent (N) was calculated for all four ODFs.

### 3.10. Drying Test

After SSE printing, the dosage forms need to dry before they are ready to use, which can take up to 48 h under ambient conditions depending on the formulation. To decrease the manufacturing time, a drying study was performed. Five different drying temperatures were investigated. Directly after printing, the films were placed in the oven with a set temperature (40, 60, 80, or 100 °C), and the time until dry was recorded and compared to ambient conditions. A tack test was performed at set time intervals until dry. The films were considered dry when they were easily detached from the transparency sheet. The moisture content of the films was measured to confirm complete drying. The quality of the oven-dried films was studied in regard to appearance and flexibility. The smoothness of the film surface and the presence of air bubbles were evaluated.

### 3.11. ATR-FTIR

The solid state of the raw materials, physical mixture 1 (PRE and HPC), physical mixture 2 (PRE, HPC and LP), physical mixture 3 (HPC and LP), and the prepared dosage forms was investigated using attenuated total reflectance Fourier transform infrared (ATR-FTIR) spectroscopy (Spectrum Two, PerkinElmer Inc., Buckinghamshire, UK). To attain a good signal during measurement, a force of 75 N was applied to the samples placed on the diamond. Samples were measured from 4000 cm^−1^ to 400 cm^−1^ with 4 accumulations at a resolution of 4cm^−1^. The measurement was performed twice on each sample; in cases where differences were observed, a third measurement was performed. The software Spectrum (version 10.03.02, PerkinElmer, Buckinghamshire, UK) was used for the acquisition of the spectra and for further data treatment, utilizing baseline correction, normalization, and data tune-up.

### 3.12. DSC

To evaluate the thermal properties of the raw materials, physical mixture 1 (PRE and HPC), physical mixture 2 (PRE, HPC, and LP), physical mixture 3 (HPC and LP), and the prepared dosage forms, differential scanning calorimetry (DSC) was utilized using the Q2000 (TA Instruments, New Castle, DE, USA). Weighed samples of 3.0 ± 0.5 mg were placed in Tzero aluminum pans, sealed, and measured with the DSC from 40 °C to 260 °C with a heating rate of 10 °C/min. Two measurements were run for each sample, and a third measurement was performed if differences were observed during the first two runs. Nitrogen with a flow rate of 50 mL/min was used as the purge gas during all measurements. The data were analyzed utilizing the TA Universal Analysis software (version 4.5A, TA Instruments).

## 4. Results and Discussion

### 4.1. Manufacturing of Veterinary Dosage Forms

#### 4.1.1. Printing Ink

**Figure 2:**
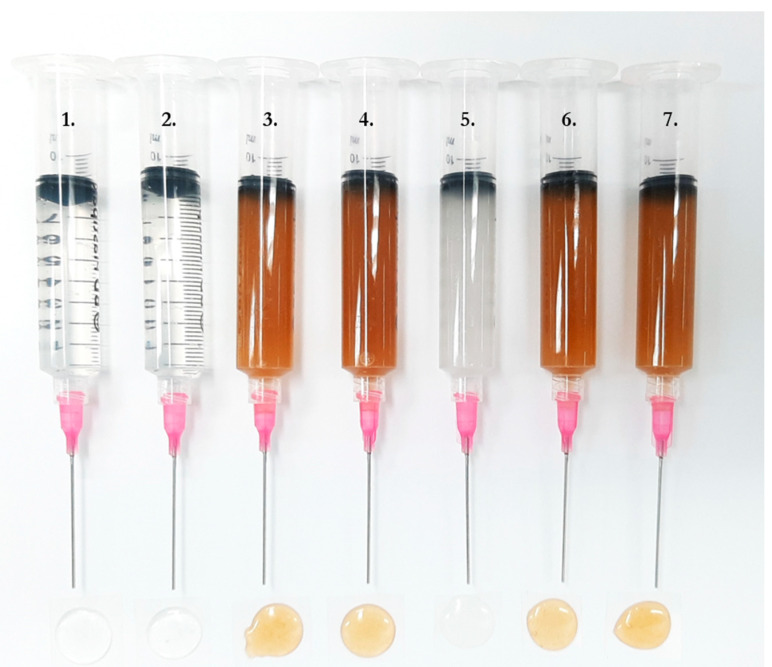
Solution-filled syringes attached with 1.5-inch 20 G precision dispensing tips and their extrudates. (1) Unloaded solution, (2) drug-loaded solution, (3) unloaded solution with liver powder, (4) drug-loaded solution with liver powder, (5) 2% drug-loaded solution, (6) nonprintable unloaded solution, and (7) nonprintable drug-loaded solution.

Three different polymers were investigated for the production of the printing ink. In the test settings, polyethylene oxide (PEO) and hydroxypropyl cellulose (HPC) LF exhibited problems dissolving into a smooth ink. HPC EXF was chosen due to its excellent film-forming capacity, degradability, and biocompatibility [36] which produced a visually homogenous, easily extrudable ink suitable for semi-solid extrusion (SSE) 3D printing. The polymer percentage needs to be high enough to produce a thick solution that keeps the extruded shape, but low enough to be printable with the SSE 3D printers with 1.5-inch 20 G tips. Drug percentages of 2% and above resulted in crystallization of the drug out of the films. In Figure 2, it can be seen that the 2% DL solution (solution 5) is cloudy due to undissolved prednisolone particles. Upon drying, orodispersible films (ODFs) printed with this solution became brittle and possessed decreased handleability. Further, due to the drug not being fully immersed in the polymer matrix, the drug crystals may fall off the films while handling, consequently, negatively affect the dose homogeneity and content uniformity. The chosen formulation, therefore, consisted of 1% prednisolone (PRE) and 25% HPC EXF in a 1:1 ratio solution with purified water and ethanol (MQ:EtOH), which resulted in a clear solution (solution 2 in the figure) and clear, flexible films without the need for plasticizers. The addition of liver powder (LP) reduced the printability, and therefore the polymer amount was decreased when LP was added. Thus, the second chosen formulation consisted of 1% PRE, 1% LP, and 24% HPC EXF in 1:1 MQ:EtOH. The addition of LP gave the solutions a rich brown color, which can be seen in the figure. Drug-loaded (DL) solutions with and without LP were printed to be analyzed, as well as unloaded (UL) solutions with and without LP, which were printed as a reference. These four solutions are henceforth referred to as DL solution, DL solution with LP, UL solution, and UL solution with LP, respectively.

#### 4.1.2. Rheology

**Figure 3:**
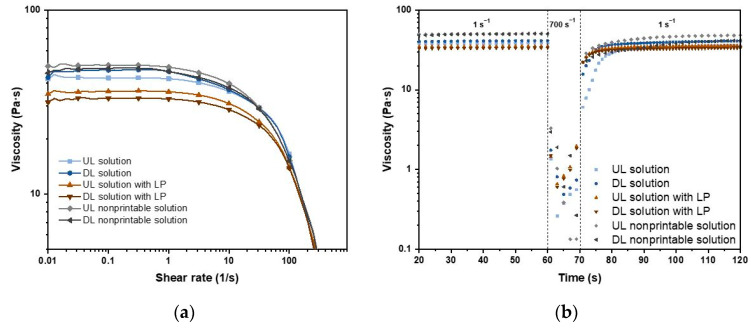
(**a**) Viscosity vs. shear rate curves of the formulations and (**b**) thixotropic behavior for the formulations sheared at 1 s^−1^ for 60 s, 700 s^−1^ for 10 s, and 1s^−1^ for 120 s.

The rheological behaviors of the four solutions mentioned above, as well as the UL and DL solutions consisting of 1% LP and 25% HPC EXF (nonprintable UL and DL solutions), were analyzed to evaluate their printability. As seen in Figure 3a, the viscosity curves of the HPC-based formulations showed Newtonian fluid behavior at a low shear rate, where the viscosity is independent of the shear rate. However, when the shear rate was increased to 10 s^−1^, the formulation exhibited a shear-thinning property, which validated the extrudability of the HPC-based formulations for SSE 3D printing. Moreover, the incorporation of PRE and LP showed a minimal effect on the overall rheological performance of the HPC-based formulations.

The viscosity of the UL solution increased after adding 1% PRE or LP. This might be due to the fact that the active pharmaceutical ingredient (API) and the LP are uncharged fractions, which did not affect the interaction between the HPC molecular chains and the solvent. In addition, the incorporation of PRE or LP increased the solid fraction of the solution/suspension, which led to an increase in the viscosity. Moreover, as LP has a larger spatial scale and more irregular shape than PRE, this hindered the interaction of the HPC molecular chains, which resulted in the increase of the zero-shear viscosity. However, incorporation of 1% PRE into the UL solution with LP resulted in a decrease in viscosity; this might be owing to the small PRE acting as a lubricant between the HPC and LP molecules during shearing, which led to a decrease in viscosity. As shown in Figure 3a, the viscosity of the HPC-based formulations is greatly affected by the concentration of HPC. The viscosity of 24% HPC formulations is lower than the 25% HPC formulation, whether mixed with 1% LP or not.

In addition, the ability to maintain the structure after extrusion is also important for SSE 3D printing. The time-dependent viscosity curves of the HPC-based formulations are displayed in Figure 3b. The high shear rate was set at 700 s^−1^ to simulate the condition of the formulations being extruded out of the nozzle. As shown in the figure, the UL solution showed the slowest viscosity recovery speed. The viscosity recovery speed was enhanced with the incorporation of PRE and LP. Overall, the HPC-based formulations had a fast viscosity recovery speed that regained 75% of their maximum viscosity in 10 s, thus ensuring their printability for SSE 3D printing.

#### 4.1.3. Semi-Solid Extrusion 3D Printing

UL and DL ODFs were successfully printed with three different SSE 3D printers according to the pre-made designs. The three printers were also evaluated for their usability. The Bocusini printer is the simplest to use, but with no modification possibilities regarding printing speed or pressure, making the personalization of doses unfeasible. The prepared ink must be manufactured to fit the printer as there are no settings to set relating to ink properties. It could be visually observed that while printing, the Bocusini printer does not equally print the six designed squares. For every square, slightly more material was printed than in the one before. The Bocusini printer could not be used in a pharmacy or animal clinic setting for the production of accurate drug dosage forms. The Zmorph printer is a three-in-one printer, where one of the functionalities is SSE 3D printing with the thick paste extruder. The original thick paste extruder was made for 100 mL syringes with no luer lock function. The use of a small tip while SSE 3D printing provides higher precision, and therefore the ZMorph printer was altered to fit a 50 mL syringe with luer lock function to enable the attachment. In the Voxelizer 2 software, there were several settings that could be adjusted, like the thickness of the gel/paste, the layer count and height, the path width, the travel and printing speed, and retraction. Problems were encountered with the extrusion of the ink, where the plunger unevenly rotated the ink, resulting in uneven extrusion of the ink and therefore uneven final films. Based on this study, the Zmorph printer would not work in a pharmacy or animal clinic setting. However, with some engineering adjustments, the Zmorph printer could potentially yield better results. The final printer, the Biobot printer, has the ability to change the pressure, travel, and print speed, the pattern of printing, layer count and height, infill, and overlap, among other parameters. The Biobot printer has the ability to print many different gels/pastes, and the settings can be adjusted to fit the properties of the ink. The main problem with the Biobot printer is scale-up, as the syringe used is only 10 mL, and some difficulties in upholding set pressure have been encountered.

The Biobot printer turned out to be the superior candidate to be use in a pharmacy or animal clinic setting when compared to the two other printers due to the even result of the extruded films that were obtained with the Biobot printer. Extemporaneous personalized medicine requires the ability to print small batches of a specific drug amount in real time for a specific patient [37]; hence, the manufacturing time of six squares for each printer was recorded. The printing times for printing six squares in one print were 5 min 40 s, 10 min 26 s, and 13 min 44 s for the Bocusini, Biobot, and Zmorph printers, respectively. For the second print, the *Y*-axis of the film design was adjusted to acquire Biobot-printed DL films of different sizes. To increase the printing time, the tip was exchanged for a shorter one, which provided the opportunity to increase the printing speed and decrease the printing pressure. The printing time was decreased from 10 min 26 s to 5 min 52 s.

### 4.2. Weight, Thickness, and Drug Amount

ODFs printed with the three different semi-solid extrusion 3D printers were studied in regard to content uniformity within and between batches. A 1% prednisolone ink (DL solution) was printed with a Bocusini, a Biobot, and a Zmorph printer. The printing was performed in batches, and for each printer, two sets of six squares were printed at a time and considered as batch 1.1 and 1.2. The printer was then restarted, and two new sets, batch 2.1 and 2.2, were printed. This was repeated once more, giving batch 3.1 and 3.2. When dried, the drug amounts in all 12 ODFs per batch were evaluated by UV-Vis. In Table 3, it can clearly be seen that the Biobot printer resulted in ODFs with the most even weight and thickness, and most importantly, high content uniformity both within and between batches with the most stable drug amount and the lowest standard deviations. The films obtained by printing with the Bocusini printer varied greatly in weight, thickness, and drug amount both within the batch and between batches. When printing, it was clearly seen that the amount of printed mass increased for each square, resulting in high standard deviations. The altered Zmorph printer had difficulties extruding the ink in a constant manner due to the circling motion of the stepper motor, resulting in high variations in weight, thickness, and drug amounts. A strong correlation (R ≥ 0.92) between the measured weight and the obtained drug amount of the prepared ODFs was found, indicating that the prepared solutions were homogenous regarding drug distribution. The obtained variations in drug amount between the ODFs was caused by the unsteady extrusion of material by the printers.

The uniformity of content (UC) was calculated on all half batches for each printer and determined for the full batches and the whole print set. To meet the UC requirements, no more than three films can be outside the 85–115% range of average content, and to meet the acceptance values (AV) requirements, the AV (L1) should be ≤15.0. In Table 3, the maximum drug content deviation from the average and the acceptance values with a target dose of 5 mg are displayed. All three whole batches printed with the Bocusini printer failed to comply with the UC and AV requirements. Only one whole batch printed with the Zmorph printer complied with the requirements of UC and AV. The Biobot printer was the only printer that fully met the UC and AV requirements for all three batches and was therefore chosen as the printer for further studies.

The weight, thicknesses, and obtained drug amounts of the different sized ODFs from the second print printed with the Biobot printer can be seen in Table 4. The two smallest films had a greater thickness due to surface tension compared to the three biggest films that had an equal thickness of 0.27 mm. All films printed with the Biobot printer showed small standard deviations regarding weight, thickness, and drug amount. A good correlation of R^2^ = 0.9934 between the obtained drug amount and the designed size of the films is observed, making it possible to personalize the doses by altering the design, and thus showing that SSE 3D printing is a suitable method for the production of personalized ODFs. A correlation value of R^2^ = 0.9972 between the weight and size of the film was obtained, which can be utilized for quality control. Small standard deviations and good correlations make the Biobot printer a potential candidate to be used in a pharmacy or hospital setting to extemporaneously produce drug dosage forms with accurate drug amounts.

### 4.3. Appearance of Dosage Forms

**Figure 4:**
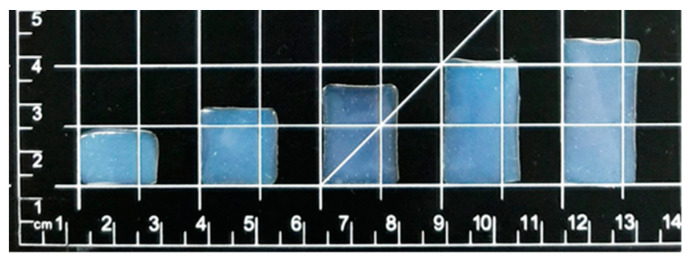
A picture of the different sized drug-loaded films without liver powder printed with the Biobot printer.

**Figure 5:**
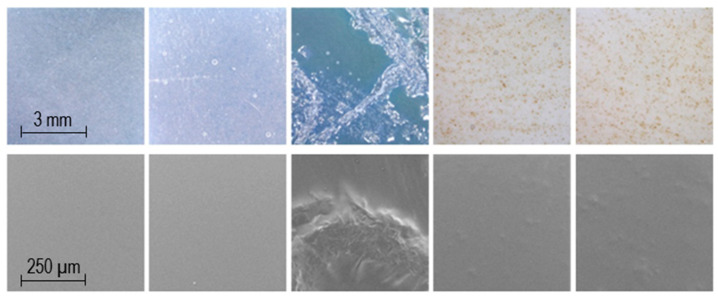
From the left, unloaded film, drug-loaded film, 2% drug-loaded film, unloaded film with liver powder, and drug-loaded film with liver powder. The upper row is taken with a handheld digital microscope, and the lower row displays scanning electron microscopy (SEM) images.

Smooth, flexible, transparent rectangular films with escalating sizes were obtained by Biobot SSE 3D printing (Figure 4). Films without LP are smooth, transparent, and have a blueish tone, which can be seen in Figure 4 and in the top two pictures from the left in Figure 5. The addition of LP altered the film color to brown, and liver particles are both tangible and visible in the films (top two pictures from the right in Figure 5). When printing a 1% PRE solution, no drug crystals were visible in the films, but when printing a 2% PRE solution, visible drug crystals emerged upon drying, which can be seen in the top middle picture in Figure 5. SEM pictures were obtained, and the same characteristics were observed as in the microscopy pictures. As can be seen in the bottom row of Figure 5, the UL and DL films without LP have a smooth surface, the 2% PRE film shows drug crystals, and the UL and DL films with LP have a rough surface due to LP particles.

### 4.4. Mechanical Properties

The mechanical properties of the films were investigated to ensure that the films had sufficient strength to be handled prior to administration [38]. Three different mechanical tests were performed, namely: puncture test, tensile strength, and folding endurance. The puncture test was performed by measuring the force it took for a ball probe to rupture a 20 × 20 × 1 mm film placed in the film support rig. The tensile force and elongation of break was obtained from a tensile test that was performed by placing a 100 × 10 × 1 mm film between the clamps, leaving 80 × 10 × 1 mm exposed, and measuring the force needed to pull the film apart as the upper clamp moves away from the stationary lower clamp. The third mechanical test performed on the films was the folding endurance test. A 20 × 20 × 1 mm film was repeatedly folded in the same spot, and the number of folds until the film cracked and broke was counted.

**Figure 6:**
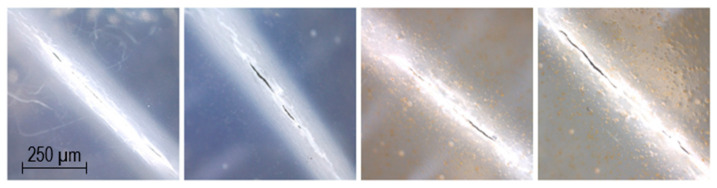
Pictures taken with a handheld digital microscope of cracks retained during the folding endurance test. From the left: unloaded film, drug-loaded film, unloaded film with liver powder, and drug-loaded film with liver powder.

Table 5 displays the measured burst strength representing the maximum tolerated force (N) on the film before rupturing, as well as the burst distance (mm) representing the flexibility of the ODF, as obtained from the puncture test measured on the second day of drying. The addition of PRE slightly weakened the film from 42.4 ± 1.0 N to 35.3 ± 0.7 N for films without LP and from 29.1 ± 0.8 N to 26.0 ± 1.2 N for films with LP. This is a common phenomenon that has been seen in several studies [15,16]. The burst strength was significantly reduced by the addition of LP to the formulation due to undissolved particles present in the film. The flexibility of the films was not affected by the addition of the drug, but the addition of LP caused a slight decrease in the flexibility of the ODFs. All the films had a high burst strength and a sufficient distance at burst, indicating that no problems should be encountered with handling the ODFs. The obtained results from the tensile strength test are also displayed in Table 5. The percentage of extension is calculated from the original length of 80 mm and the elongation at break. A high percentage of extension equals higher flexibility. The tests show that the more solid material present in the film, the lower the mechanical strength. The UL films without LP had the highest maximum forces of breakage, followed by the DL film without LP, the UL with LP, and lastly, the DL film with LP. In the tensile test, the maximum stretch distance was slightly lower with the presence of PRE and/or LP. A percentage of elongation >10% is preferable for sufficient handling properties [30]. All ODFs showed adequate tensile strength with a percentage of elongation higher than 10%. According to the folding endurance test, the DL film without LP could withstand the highest number of folds, cracking at 40 folds and breaking at 46 folds. UL films without LP could also withstand a substantial number of folds, but less than the DL film without LP, indicating that prednisolone could contribute to a plasticizing effect. Both the UL and DL films with LP were very sensitive to folds; the UL film cracked after the first fold, while the DL film could withstand six folds without cracking. Both films broke at eight folds. In Figure 6, microscopy pictures of the broken films can be seen. To pass a folding endurance test and show good flexibility, the number of folds should be greater than 300 folds [32,39]. All the prepared films failed to comply with this standard, but all ODFs endured handling well without any damage, fulfilling the European Pharmacopoeia requirements for ODFs [40].

### 4.5. Moisture Content

Moisture content measurement was performed using a moisture analyzer, where a sample of 0.5 g was heated up to 120 °C and the mass-% weight loss corresponding to moisture evaporation was recorded. The measurement was performed in triplicate. In the literature, regardless of polymer or drug substance, the moisture content of the studied films has generally been less than 10% [30,41,42]. According to Nair et al. [43], the moisture content of buccal films should be <5%. In the present study, both UL and DL films with and without LP had a moisture content of 3.0 ± 0.4, 3.2 ± 0.5, 3.2 ± 0.1, and 3.1 ± 0.2%, respectively, which are all very similar and well within the recommended limits. As seen in the mechanical tests, the films were still flexible despite having a relatively low moisture content. Neither the presence of LP nor the drug seemed to affect the moisture content of the films. The storage conditions are particularly crucial for ODFs. With HPC being a hygroscopic excipient [26], there is a possibility of water sorption over time. Besides the risk of microbial contamination, it can possibly affect the stability of prednisolone and alter the mechanical properties of the films. It is essential to store the dried ODFs in airtight containers at relatively low humidity.

### 4.6. Surface pH

The surface pH of an ODF should preferably be as close to physiological saliva (5.8–7.4) as possible to ensure a comfortable mouthfeel. Local mucosal irritation and discomfort may occur with the administration of an ODF with a pH outside this range [44]. In this study, the surface pH was determined by adding 1 mL of MQ on top of the film sample, bringing the electrode to the solution after 30 s, and measuring the pH after allowing equilibration for 1 min. UL and DL films without LP had a surface pH of 4.83 ± 0.15 and 4.63 ± 0.65, respectively, which is too acidic for an ODF and may cause discomfort upon administration. The presence of prednisolone slightly lowered the pH compared to the UL films, which could be seen for both films with and without LP. The addition of LP revealed a significant increase in the pH, bringing it to neutral levels, giving the UL films with LP a surface pH of 7.17 ± 0.29 and the DL films with LP a surface pH of 6.59 ± 0.07, which are in the range of physical saliva and should not cause local side effects at the administration site.

### 4.7. Adhesiveness

Mucoadhesiveness is an important property of orodispersible films; the film needs to stick to the mucosa and not float around in the mouth. A non-adhesive film is easier for the animal to spit out, leading to treatment failure. The most relevant parameters to study are the adhesion force and the work of adhesion [43]. As Woertz et al. [44] point out, there are different methods for evaluating mucoadhesion. The comparability of test results is challenging because of the many different methods, test setups, and parameters. The mucoadhesion test performed in this study did, however, give an idea of how the adhesiveness is affected by the film composition. The test was performed by attaching a 10 × 10 × 1 mm piece of film to a cylindrical probe attached to a texture analyzer and bringing the probe down to touch the hydrated artificial skin attached to the film support rig. The force required to detach the film piece from the artificial skin after 10 min of contact was measured. The sample pieces used in the mucoadhesion test were smaller than the final ODFs. In reality, the ODF would probably adhere better to the mucosa because of the larger contact/wetting surface. As seen in Table 6, all ODFs had similar adhesiveness, work of adhesion, and distance of travel.

### 4.8. Disintegration

The fast disintegration of ODFs is crucial due to the nature of the dosage form European Pharmacopoeia does not include any specific method or acceptance limits for testing the disintegration behavior of ODFs. Generally, the limit set for the disintegration time of orodispersible tablets (ODTs) (less than 3 min) has been used for ODFs as well [30,45]. Setting up a disintegration test that yields comparable and relevant results is challenging. A key factor in the disintegration of an ODF is the texture, movement, and pressure of the tongue; these are hard to mimic in laboratory settings. The test was performed using an in-house-made slide frame and ball measurement device. Three films (30 × 40 × 1 mm) were placed between the frames, 100 µL of MQ and a steel ball were placed in the middle of each film, and a further 3 mL of MQ was added. The apparatus was placed in a shaking incubator, and the time it took for the ball to break the film was recorded. In this study, the disintegration times for the UL film, DL film, UL film with LP, and DL film with LP were 08:20 ± 03:16, 13:30 ± 04:37, 03:12 ± 00:17, and 03:32 ± 00:30 min, respectively. The films without LP, both UL and DL, had a longer disintegration time with high standard deviations. This is due to the gelification of the HPC when it comes in contact with water. The addition of LP and, therefore, the presence of particles in the film, decreased the disintegration time to almost comply with the disintegration limit described for ODTs.

### 4.9. In Vitro Dissolution

**Figure 7:**
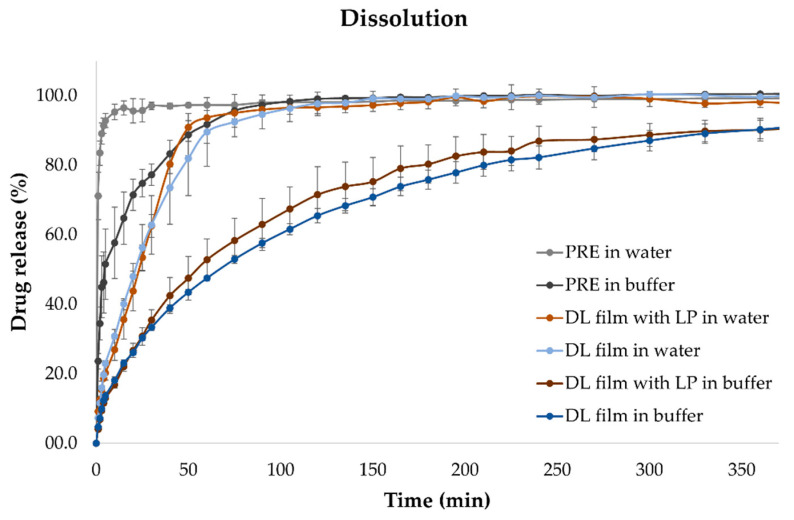
Drug release of pure substance and drug-loaded films with and without liver powder in phosphate buffer (pH 6.8) and in water. Average and standard deviations are shown, *n* = 3.

Drug release studies were performed in both phosphate buffer (pH 6.8) and in water. For in vitro dissolution studies, the samples were placed in vessels with 500 mL of media. The test was performed in a dissolution bath and aliquots of media were automatically withdrawn and measured at predefined time-points with an online UV-Vis spectrophotometer. As can be seen in Figure 7, pure substance prednisolone releases rapidly in water and is fully dissolved within minutes. PRE has a slower dissolution in buffer, and it takes up to 40 min for pure PRE to have an 80% release. The drug release of the DL film without LP in water was slightly slower compared to the pure substance at first due to the gelation of the HPC, but the ODF rapidly dissolved and reached 80% drug release within 50 min. In buffer, the drug release of PRE from the ODFs is slow due to potential cross-linking of the polymer, and only 90% drug release is achieved. Films containing LP have a slightly faster drug release profile due to the presence of solid particles, leading to a faster disintegration time in the disintegration study. When performing drug release studies on HPC films in buffer, the film does not entirely dissolve; thus, an immersion study was conducted to investigate the behavior of HPC films in water versus buffer.

**Figure 8:**
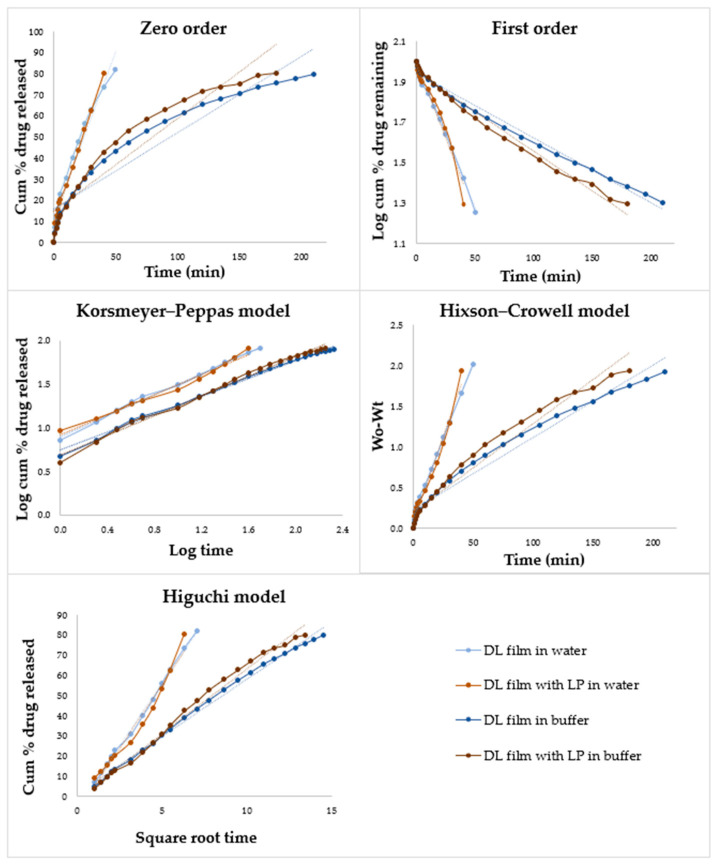
Drug release data fitted to various kinetic models (zero order, first order, Korsmeyer–Peppas (Kors–Peppas), Hixson–Crowell (Hixson), and Higuchi) obtained from the dissolution studies of the drug-loaded films with and without liver powder in water and in buffer.

The dissolution data up to 80% drug release for DL ODFs with and without LP in water and in buffer were fitted to zero order, first order, Korsmeyer–Peppas, Hixson–Crowell, and the Higuchi drug diffusion models, as can be seen in Figure 8. In Table 7, the obtained correlation coefficients (R^2^) for the different models and the calculated release exponent (N) values are displayed. All ODFs showed a high R^2^ for the Korsmeyer–Peppas drug diffusion model and had N values above 0.5, indicating that the release mechanism is non-Fickian diffusion, which indicates that the drug is released from the dosage form by diffusion and swelling, and that the release is time-dependent. The drug usually releases from a HPC matrix by swelling of the HPC, leading to erosion and diffusion from the gel layer [46]. The drug release of the DL films in water showed good correlation (R^2^ < 0.99) for all the models except the zero order. The addition of LP to the film gave the ODF a zero order release kinetic in water. DL ODFs with and without LP followed a Higuchi release kinetic in buffer.

### 4.10. Drying Time

**Figure 9:**
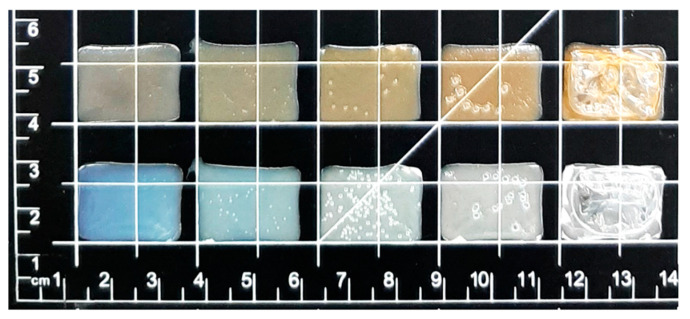
Pictures of drug-loaded films with liver powder (upper row) and drug-loaded films (lower row). From the left, after drying at room temperature, 40, 60, 80, and 100 °C.

**Figure 10:**
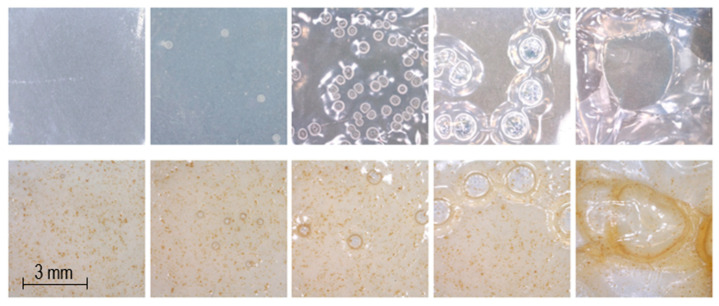
Microscopy pictures of drug-loaded films (upper row) and drug-loaded films with liver powder (lower row). From the left, after drying at room temperature, 40, 60, 80, and 100 °C. Taken with a handheld digital microscope.

The most time-consuming step in the manufacturing of the ODFs is the drying of the final dosage form. The use of elevated temperatures to decrease manufacturing times was therefore explored. The films were dried at room temperature and in ovens set to 40, 60, 80, or 100 °C; the time needed for the film to dry was recorded, and the appearance and flexibility were evaluated. It took 24 h for the films to dry at room temperature, resulting in flexible films without bubbles. When drying in elevated temperatures, the drying time was decreased, but bubbles became present in the films. The chosen time and temperature to dry the films in was 5 h at 40 °C, which produced flexible films with few bubbles present. The higher the temperature, the lower the drying time, resulting in 3, 2, and 1 h of drying at 60, 80, and 100 °C, but the films were bubbly and uneven (see Figure 9 and Figure 10) and became hard and brittle. In Figure 9, a slight shift in the color can be seen. The presence of water tends to give the films a blue color. With drying, this color fades, which could be due to changes in the reflection of light.

### 4.11. ATR-FTIR

**Figure 11:**
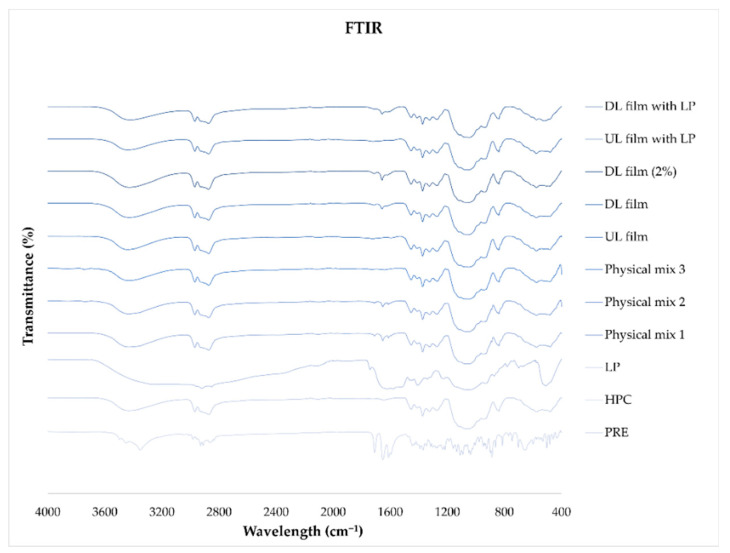
FTIR spectra of pure substances, physical mixtures, and unloaded and drug-loaded films with and without liver powder. Physical mixture 1 is a mixture of prednisolone and hydroxypropyl cellulose, physical mixture 2 contains all three dry substances (prednisolone, hydroxypropyl cellulose, and liver powder), and physical mixture 3 is a blend of hydroxypropyl cellulose and liver powder.

The ATR-FTIR spectra of the raw material, physical mixtures, and the UL and DL films with and without LP were measured from 4000 cm^−1^ to 400 cm^−1^ with four accumulations at a resolution of 4 cm^−1^ and an applied force of 75 N. The results are presented in Figure 11. Pure PRE displayed characteristic peaks at 3497 cm^−1^, 3453 cm^−1^, and 3350 cm^−1^ of the O–H stretch vibration; the presence of carbonyl groups in its structure was shown in the characteristic peaks at 1711 cm^−1^ and 1652 cm^−1^ [47], and the peak at 1612 cm^−1^ showed the presence of aromatics [48]. The broad band at 3600–3100 cm^−1^ for the pure HPC spectrum, with a maximum at 3450 cm^−1^, corresponds to the stretching vibration of the –OH groups [49]. An absorption band at 2970 cm^−1^ is due to CH_2_ stretching vibration, and the broad absorption band at 1074 cm^−1^ is due to C–O–C stretching vibration in HPC [50]. In the DL films, the broad peak of HPC at 3600–3100 cm^−1^ hides any characteristic peaks of PRE in that area. The carbonyl groups in PRE are present in both the physical mixtures and the DL films and can be found at 1712–1708 cm^−1^ and 1657–1651 cm^−1^. These peaks are more profound in the drug-loaded film with 2% PRE compared to the 1% PRE-loaded film, indicating that infrared spectroscopy could be utilized in quality assurance. The presence of LP in the physical mixtures or films with LP is not evident in the spectra.

**Figure 12:**
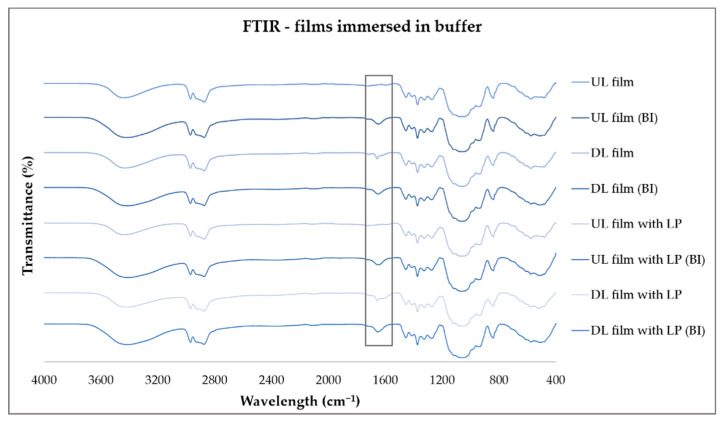
FTIR spectra of dried and buffer-immersed (BI) dried unloaded and drug-loaded films with and without liver powder.

During the dissolution studies, it was observed that the HPC films did not completely dissolve in the buffer and instead became opaque. An immersion study was performed to study this behavior by placing the prepared films in both room temperate buffer and 37 °C buffer for 12 h. After 12 h, the films were placed to dry, and the solid state was investigated by FTIR. Films immersed in buffer at room temperature completely dissolved, but in elevated temperatures, the immersed films remained intact and turned opaque. In Figure 12, the FTIR spectra of the dry films before immersion in buffer and of films immersed in 37 °C buffer for 12 h and dried prior to measurement are displayed. The dried buffer-immersed (BI) films all display a peak at around 1650 cm^−1^ that is not present for the non-immersed films. Phosphate can be used as a cross-linking agent of cellulose and elevated temperatures can aid the process. The observed new peak could be due to the cross-linking of HPC or the presence of water. Specific phosphate groups found at 1298 cm^−1^ and 997 cm^−1^ ascribed to P=O and P–O, respectively, could not be found [51].

### 4.12. DSC

**Figure 13:**
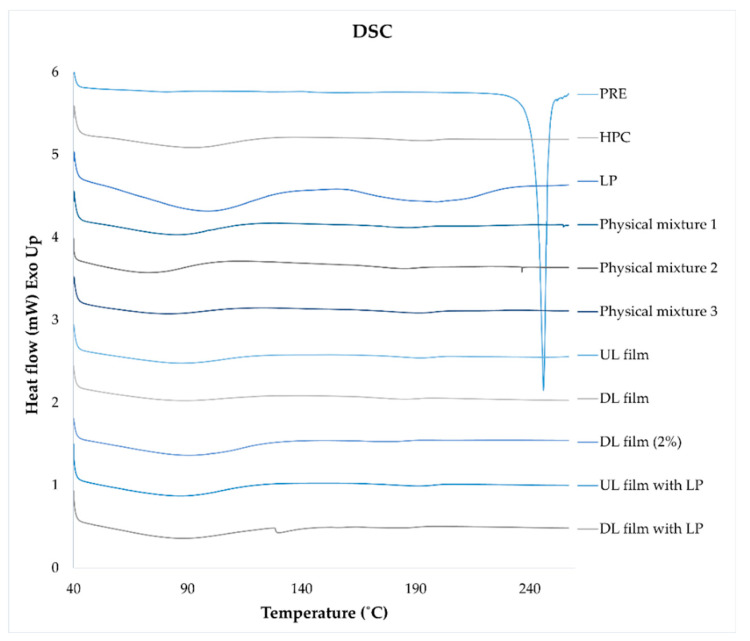
DSC curves of pure substances, physical mixtures, and unloaded and drug-loaded films with and without liver powder. Physical mixture 1 is a mixture of prednisolone and hydroxypropyl cellulose, physical mixture 2 contains all three dry substances (prednisolone, hydroxypropyl cellulose, and liver powder), and physical mixture 3 is a blend of hydroxypropyl cellulose and liver powder.

For thermal evaluation, weighed samples of 3.0 ± 0.5 mg were placed in Tzero aluminum pans, sealed, and measured with the DSC from 40 °C to 260 °C with a heating rate of 10 °C/min. In Figure 13, the DSC curves of the pure substances, the physical mixtures, and the prepared films can be seen. A slight observation of the crystalline melt of HPC can be observed for the pure substance of HPC, the physical mixtures, and the prepared films at 165–210 °C, which is in line with the literature [52]. Evaporation of water is noticeable at around 100 °C for most of the samples. Pure substance PRE shows a melting onset at 231 °C with a melting peak at 246 °C [53]. A small peak at 238 °C can be seen for physical mixture 2, but the prepared drug-loaded films do not show a melting point of PRE, indicating that the drug is in an amorphous state in the ODFs.

## 5. Conclusions

Semi-solid extrusion 3D printing is a fairly simple 3D printing technology with many advantages. The printing does not require high temperatures and suits many different drug substances. The required excipients are few, and the ink preparation is undemanding, thus making SSE 3D printing a suitable technology to be used in a pharmacy or veterinary clinic setting. Prednisolone-containing orodispersible films were successfully printed utilizing SSE. The Biobot bioprinter produced uniform ODFs with a great correlation between the film design and the drug amounts, which met the CU and AV requirements of European Pharmacopoeia, indicating that accurate, personalized doses can be achieved utilizing this printer. Critical quality attributes for ODFs are fast disintegration, high mechanical strength to withstand handling, neutral pH, and low moisture content. The prepared ODFs possessed good flexibility and high strength and did not suffer any damage while being handled. All films had a low moisture content well below the suggested limit. The addition of the taste-enhancer LP increased the pH to a neutral level and increased the disintegration time and the dissolution of the ODFs. The manufacturing time of the ODFs could be reduced by oven-drying the films at 40 °C for five hours. This study showed that SSE is a promising technique for the extemporaneous manufacturing of prednisolone-containing ODFs for veterinary use. In an area where there is a high demand for personalized doses, this technique could provide a solution.

## Figures and Tables

**Table 1:** Results obtained of the potency ranges of extemporaneously manufactured drug products prepared between the years 2006–2019. The tests were conducted by The Missouri Board of Pharmacy.

Year	Tests Performed	Unsatisfactory Percentage (%)	Potency Range (%)
2019	55	36.4	53.7–193.9
2018	72	23.6	64.4–175.3
2017	80	27.5	66.0–235.2
2016	83	22.9	37.6–155.8
2015	58	22.4	13.4–258.0
2014	70	18.6	64.6–156.7
2013	56	12.5	3.3–226.6
2012	63	11.1	3.3–226.6
2011	158	17.1	8.3–196.1
2010	225	15.1	28.0–197.2
2009	242	11.6	0.0–145.2
2008	186	24.7	21.3–373.7
2007	213	23.9	21.2–450.4
2006	274	25.2	0.0–259.0

**Table 2:** Content of the unloaded and drug-loaded printable and nonprintable solutions with and without liver powder.

Solution	PRE (%)	LP (%)	HPC (%)	Solvent
UL solution	-	-	25	1:1 MQ:EtOH (*v/v*)
DL solution	1	-	25	1:1 MQ:EtOH (*v/v*)
UL solution with LP	-	1	24	1:1 MQ:EtOH (*v/v*)
DL solution with LP	1	1	24	1:1 MQ:EtOH (*v/v*)
2% DL solution	2	-	25	1:1 MQ:EtOH (*v/v*)
UL nonprintable solution	-	1	25	1:1 MQ:EtOH (*v/v*)
DL nonprintable solution	1	1	25	1:1 MQ:EtOH (*v/v*)

UL: unloaded; DL: drug-loaded; PRE: prednisolone; LP: liver powder; HPC: hydroxypropyl cellulose; MQ: purified water; EtOH: ethanol.

**Table 3:** Weight, thickness, drug amount, the maximum deviation (Max Dev) of content uniformity, and uniformity of dosage units (acceptance value (AV)) of all the printed batches. Six doses were printed and measured for each half batch, and the average (AVG) and standard deviations were calculated for each half batch, batch, and all batches together for each printer.

Sample	Weight (mg)	Thickness (mm)	Drug Amount (mg)	Max Dev (%)	AV
Bocusini batch 1.1	60.8 ± 11.4	0.23 ± 0.04	5.3 ± 0.8	−34.8b **	44.7d
Bocusini batch 1.2	66.4 ± 9.7	0.24 ± 0.03	5.8 ± 0.6	−21.4a *	43.8d
Bocusini batch 2.1	68.2 ± 8.6	0.24 ± 0.03	6.0 ± 0.7	−19.4a *	52.6d
Bocusini batch 2.2	69.8 ± 9.1	0.25 ± 0.02	6.0 ± 0.9	−26.5b *	63.4d
Bocusini batch 3.1	87.7 ± 5.3	0.30 ± 0.01	7.4 ± 0.4	33.0b ***	65.2d
Bocusini batch 3.2	71.9 ± 9.1	0.26 ± 0.02	6.3 ± 0.7	15.5e **	55.8d
Bocusini batch 1 AVG	63.6 ± 10.5	0.23 ± 0.03	5.5 ± 0.7	−34.8b ***	44.8d
Bocusini batch 2 AVG	69.0 ± 8.5	0.25 ± 0.02	6.0 ± 0.8	−26.5b **	56.4d
Bocusini batch 3 AVG	79.8 ± 10.9	0.28 ± 0.03	6.8 ± 0.8	33.0b ***	72.2d
Bocusini AVG	70.8 ± 11.9	0.26 ± 0.04	6.1 ± 0.9	−34.8b ***	65.0d
Biobot batch 1.1	65.1 ± 0.1	0.25 ± 0.01	4.9 ± 0.1	6.6a	4.6c
Biobot batch 1.2	65.1 ± 0.3	0.26 ± 0.01	4.8 ± 0.1	3.1a	6.2c
Biobot batch 2.1	63.4 ± 0.2	0.24 ± 0.01	4.7 ± 0.1	2.4a	6.6c
Biobot batch 2.2	60.9 ± 1.2	0.24 ± 0.01	4.4 ± 0.1	−8.4a	13.9c
Biobot batch 3.1	63.3 ± 0.1	0.25 ± 0.01	4.7 ± 0.1	2.0a	8.4c
Biobot batch 3.2	63.1 ± 0.2	0.23 ± 0.01	4.6 ± 0.1	−5.5a	12.2c
Biobot batch 1 AVG	65.1 ± 0.2	0.25 ± 0.01	4.8 ± 0.1	6.6a	6.5c
Biobot batch 2 AVG	62.2 ± 1.6	0.24 ± 0.01	4.6 ± 0.2	−8.4a	15.0c
Biobot batch 3 AVG	63.2 ± 0.2	0.24 ± 0.01	4.6 ± 0.1	−5.5a	11.2c
Biobot AVG	63.5 ± 1.5	0.24 ± 0.01	4.7 ± 0.2	−8.4a	12.6c
Zmorph batch 1.1	63.4 ± 5.2	0.23 ± 0.01	4.7 ± 0.5	−23.2b ***	28.7d
Zmorph batch 1.2	74.9 ± 2.5	0.28 ± 0.02	5.4 ± 0.1	4.9a	13.9c
Zmorph batch 2.1	65.6 ± 1.5	0.25 ± 0.01	4.8 ± 0.2	−15.1a *	9.3c
Zmorph batch 2.2	67.8 ± 4.0	0.25 ± 0.01	5.1 ± 0.3	−15.9a *	14.8c
Zmorph batch 3.1	86.8 ± 5.9	0.33 ± 0.07	6.5 ± 0.4	30.1b ***	47.2d
Zmorph batch 3.2	75.7 ± 6.4	0.30 ± 0.03	5.6 ± 0.4	15.1b *	31.9d
Zmorph batch 1 AVG	69.2 ± 7.2	0.25 ± 0.03	5.0 ± 0.5	−23.2b ***	25.7d
Zmorph batch 2 AVG	66.7 ± 3.1	0.25 ± 0.01	4.9 ± 0.2	−15.9e **	12.2c
Zmorph batch 3 AVG	81.3 ± 8.2	0.31 ± 0.05	6.0 ± 0.6	30.1b ***	48.8d
Zmorph AVG	72.4 ± 9.1	0.27 ± 0.04	5.4 ± 0.7	30.1b ***	38.7d

Number of individual doses outside ± 15% limits: * = 1 dose, ** = 2 doses, *** ≥ 3 doses. a = complies with the requirements for UC, b = does not comply with the requirements for UC, c = complies with the requirements for AV, d = does not comply with the requirements for AV, e = an additional 20 units should be tested to reveal if the test passed or failed.

**Table 4:** Measured weight, thickness, and drug amount of semi-solid extrusion (SSE) 3D printed films.

Film Size (mm)	Weight (mg)	Thickness (mm)	Drug Amount (mg)
10 × 15 × 1	79.1 ± 1.3	0.32 ± 0.01	2.7 ± 0.1
15 × 15 × 1	98.6 ± 1.0	0.30 ± 0.01	4.1 ± 0.1
20 × 15 × 1	122.1 ± 0.6	0.27 ± 0.01	5.4 ± 0.1
25 × 15 × 1	147.6 ± 3.4	0.27 ± 0.02	6.3 ± 0.1
30 × 15 × 1	173.2 ± 2.3	0.27 ± 0.02	7.3 ± 0.1

**Table 5:** Results from the three mechanical tests (puncture test, tensile test, and folding endurance) on the unloaded and drug-loaded films with and without liver powder, *n* = 6.

Mechanical Test	UL Film	DL Film	UL Film with LP	DL Film with LP
Puncture test				
Burst strength (N)	42.4 ± 1.0	35.3 ± 0.7	29.1 ± 0.8	26.0 ± 1.2
Distance at burst (mm)	3.6 ± 0.2	3.6 ± 0.2	3.1 ± 0.1	3.2 ± 0.1
Tensile test				
Maximum tensile force (N)	33.9 ± 1.7	28.8 ± 1.4	28.0 ± 0.4	21.8 ± 0.4
Elongation of break (mm)	15.6 ± 4.2	14.1 ± 2.8	12.1 ± 1.7	10.3 ± 1.1
Percentage of extension (%)	19.5 ± 5.3	17.6 ± 3.5	15.1 ± 2.2	12.9 ± 1.4
Folding endurance				
No. of folds until cracked	27 ± 2.6	40 ± 0.0	6 ± 0.0	0 ± 0.0
No. of folds until broken	31.7 ± 2.9	46.0 ± 1.0	8.0 ± 0.0	8.0 ± 1.0

**Table 6:** The adhesiveness of unloaded and drug-loaded films with and without liver powder, measured on the second day from printing. Average and standard deviations are presented, *n* = 6.

Sample	Adhesiveness (N)	Work of Adhesion (N.s)	Travel (mm)
UL film	0.15 ± 0.02	1.26 ± 0.31	1.96 ± 0.35
DL film	0.12 ± 0.01	0.89 ± 0.12	1.65 ± 0.57
UL film with LP	0.14 ± 0.03	1.07 ± 0.17	1.72 ± 0.24
DL film with LP	0.21 ± 0.06	1.54 ± 0.71	1.62 ± 0.53

**Table 7:** In vitro release kinetic values.

Formulation	Zero Order ^1^ R^2^	First Order ^2^ R^2^	Higuchi ^3^ R^2^	Hixson ^4^ R^2^	Kors–Peppas ^5^ R^2^	N
DL film in water	0.9563	0.9958	0.9975	0.9939	0.9961	0.595
DL film with LP in water	0.9857	0.9544	0.9658	0.978	0.9852	0.580
DL film in buffer	0.9105	0.9873	0.9951	0.9687	0.995	0.518
DL film with LP in buffer	0.9072	0.9855	0.9917	0.9659	0.9928	0.588

^1^ Zero order equation: Q_t_ = Q_0_ + K_0_ t; ^2^ first order equation: ln Q_t_ = ln Q_0_ + K_1_ t; ^3^ Higuchi equation: Q_t_ = K_H_ t^½^; ^4^ Hixson–Crowell equation: Q_0_^1/3^ – Q_t_^1/3^ = Kst; ^5^ Korsmeyer–Peppas equation: Q_t_/Q_∞_ = Kkt^n^. R^2^: correlation coefficient; Q_t_: amount of drug released in time t; Q_0_: initial amount of drug in the dosage form; Q_∞_: total amount of drug dissolved when the dosage form is exhausted; K_0_, K_1_, K_H_, K_s_, K_k_: release rate constants; N: release exponent (indicative of drug release mechanism).

## References

[1] Davidson G. (2017). Veterinary compounding: Regulation, challenges, and resources. Pharmaceutics.

[2] European Pharmacopoeia Commission (2020). Pharmaceutical preparations. European Pharmacopoeia.

[3] Missouri Board of Pharmacy, Annual Reports from 2006–2019. https://pr.mo.gov/pharmacists-annual-reports.asp.

[4] Bramwell B., Pharm B.S., Williams L.A. (2009). Recommended Tips for Treating Veterinary Patients. Int. J. Pharm. Compd..

[5] Lowe A.D., Campbell K.L., Graves T. (2008). Glucocorticoids in the cat. Vet. Dermatol..

[6] Wening K., Breitkreutz J. (2011). Oral drug delivery in personalized medicine: Unmet needs and novel approaches. Int. J. Pharm..

[7] Sandler N., Preis M. (2016). Printed Drug-Delivery Systems for Improved Patient Treatment. Trends Pharmacol. Sci..

[8] Goyanes A., Buanz A.B.M., Hatton G.B., Gaisford S., Basit A.W. (2015). 3D printing of modified-release aminosalicylate (4-ASA and 5-ASA) tablets. Eur. J. Pharm. Biopharm..

[9] Öblom H., Zhang J., Pimparade M., Speer I., Preis M., Repka M., Sandler N. (2019). 3D-Printed Isoniazid Tablets for the Treatment and Prevention of Tuberculosis—Personalized Dosing and Drug Release. AAPS PharmSciTech.

[10] Goyanes A., Robles Martinez P., Buanz A., Basit A.W., Gaisford S. (2015). Effect of geometry on drug release from 3D printed tablets. Int. J. Pharm..

[11] Yu D.-G., Branford-White C., Ma Z.H., Zhu L.M., Li X.Y., Yang X.L. (2009). Novel drug delivery devices for providing linear release profiles fabricated by 3DP. Int. J. Pharm..

[12] Yu D.-G., Branford-White C., Yang Y.-C., Zhu L.-M., Welbeck E.W., Yang X.-L. (2009). A novel fast disintegrating tablet fabricated by three-dimensional printing. Drug Dev. Ind. Pharm..

[13] Khaled S.A., Burley J.C., Alexander M.R., Yang J., Roberts C.J. (2015). 3D printing of five-in-one dose combination polypill with defined immediate and sustained release profiles. J. Control. Release.

[14] Khaled S.A., Burley J.C., Alexander M.R., Roberts C.J. (2014). Desktop 3D printing of controlled release pharmaceutical bilayer tablets. Int. J. Pharm..

[15] Öblom H., Sjöholm E., Rautamo M., Sandler N. (2019). Towards printed pediatric medicines in hospital pharmacies: Comparison of 2d and 3d-printed orodispersiblewarfarin films with conventional oral powders in unit dose sachets. Pharmaceutics.

[16] Sjöholm E., Sandler N. (2019). Additive manufacturing of personalized orodispersible warfarin films. Int. J. Pharm..

[17] Vithani K., Goyanes A., Jannin V., Basit A.W., Gaisford S., Boyd B.J. (2019). A Proof of Concept for 3D Printing of Solid Lipid-Based Formulations of Poorly Water-Soluble Drugs to Control Formulation Dispersion Kinetics. Pharm. Res..

[18] Seoane-Viaño I., Ong J.J., Luzardo-Álvarez A., González-Barcia M., Basit A.W., Otero-Espinar F.J., Goyanes A. (2020). 3D printed tacrolimus suppositories for the treatment of ulcerative colitis. Asian J. Pharm. Sci..

[19] Gyles C. (2019). 3D printing comes to veterinary medicine. Can. Vet. J..

[20] Meléndez P.A., Kane K.M., Ashvar C.S., Albrecht M., Smith P.A. (2008). Thermal inkjet application in the preparation of oral dosage forms: Dispensing of prednisolone solutions and polymorphic characterization by solid-state spectroscopic techniques. J. Pharm. Sci..

[21] Vakili H., Wickström H., Desai D., Preis M., Sandler N. (2017). Application of a handheld NIR spectrometer in prediction of drug content in inkjet printed orodispersible formulations containing prednisolone and levothyroxine. Int. J. Pharm..

[22] Farto-Vaamonde X., Auriemma G., Aquino R.P., Concheiro A., Alvarez-Lorenzo C. (2019). Post-manufacture loading of filaments and 3D printed PLA scaffolds with prednisolone and dexamethasone for tissue regeneration applications. Eur. J. Pharm. Biopharm..

[23] Skowyra J., Pietrzak K., Alhnan M.A. (2015). Fabrication of extended-release patient-tailored prednisolone tablets via fused deposition modelling (FDM) 3D printing. Eur. J. Pharm. Sci..

[24] Vithani K., Goyanes A., Jannin V., Basit A.W., Gaisford S., Boyd B.J. (2019). An Overview of 3D Printing Technologies for Soft Materials and Potential Opportunities for Lipid-based Drug Delivery Systems. Pharm. Res..

[25] Rowe R.C., Sheskey P.J., Quinn M.E. (2009). Handbook of Pharmaceutical Excipients.

[26] European Pharmacopoeia Commission (2020). 2.9.6. Uniformity of content of single-dose preparations. European Pharmacopoeia.

[27] European Pharmacopoeia Commission (2020). 2.9.40. Uniformity of dosage units. European Pharmacopoeia.

[28] Preis M., Woertz C., Kleinebudde P., Org Breitkreutz J. (2013). Oromucosal film preparations: Classification and characterization methods. Expert Opin. Drug Deliv..

[29] Visser J.C., Woerdenbag H.J., Crediet S., Gerrits E., Lesschen M.A., Hinrichs W.L.J., Breitkreutz J., Frijlink H.W. (2015). Orodispersible films in individualized pharmacotherapy: The development of a formulation for pharmacy preparations. Int. J. Pharm..

[30] Lir I., Haber M., Dodiuk-Kenig H. (2007). Skin surface model material as a substrate for adhesion-to-skin testing. J. Adhes. Sci. Technol..

[31] Tejada G., Barrera M.G., Piccirilli G.N., Sortino M., Frattini A., Salomón C.J., Lamas M.C., Leonardi D. (2017). Development and Evaluation of Buccal Films Based on Chitosan for the Potential Treatment of Oral Candidiasis. AAPS PharmSciTech.

[32] Steiner D., Finke J.H., Kwade A. (2016). Efficient production of nanoparticle-loaded orodispersible films by process integration in a stirred media mill. Int. J. Pharm..

[33] European Pharmacopoeia Commission (2020). 2.9.3. Dissolution test for solid dosage forms. European Pharmacopoeia.

[34] Basak S.C., Kumar K.S., Ramalingam M. (2008). Design and release characteristics of sustained release tablet containing metformin HCl. Rev. Bras. Ciencias Farm. J. Pharm. Sci..

[35] Keshawy M., El-Moghny T.A., Abdul-Raheim A.R.M., Kabel K.I., El-Hamouly S.H. (2013). Synthesis and characterization of oil sorbent based on Hydroxypropyl Cellulose Acrylate. Egypt. J. Pet..

[36] Zema L., Melocchi A., Maroni A., Gazzaniga A. (2017). Three-Dimensional Printing of Medicinal Products and the Challenge of Personalized Therapy. J. Pharm. Sci..

[37] Szakonyi G., Zelkó R. (2012). The effect of water on the solid state characteristics of pharmaceutical excipients: Molecular mechanisms, measurement techniques, and quality aspects of final dosage form. Int. J. Pharm. Investig..

[38] Koland M., Charyulu R.N., Prabhu P. (2010). Mucoadhesive films of losartan potassium for buccal delivery: Design and characterization. Indian J. Pharm. Educ. Res..

[39] European Pharmacopoeia Commission (2020). Dosage forms. European Pharmacopoeia.

[40] Elmeshad A.N., El Hagrasy A.S. (2011). Characterization and optimization of orodispersible mosapride film formulations. AAPS PharmSciTech.

[41] Foo W.C., Khong Y.M., Gokhale R., Chan S.Y. (2018). A novel unit-dose approach for the pharmaceutical compounding of an orodispersible film. Int. J. Pharm..

[42] Nair A.B., Kumria R., Harsha S., Attimarad M., Al-Dhubiab B.E., Alhaider I.A. (2013). In vitro techniques to evaluate buccal films. J. Control. Release.

[43] Pechová V., Gajdziok J., Muselík J., Vetchý D. (2018). Development of Orodispersible Films Containing Benzydamine Hydrochloride Using a Modified Solvent Casting Method. AAPS PharmSciTech.

[44] Woertz C., Preis M., Breitkreutz J., Kleinebudde P. (2013). Assessment of test methods evaluating mucoadhesive polymers and dosage forms: An overview. Eur. J. Pharm. Biopharm..

[45] Thabet Y., Lunter D., Breitkreutz J. (2018). Continuous manufacturing and analytical characterization of fixed-dose, multilayer orodispersible films. Eur. J. Pharm. Sci..

[46] Klančar U., Baumgartner S., Legen I., Smrdel P., Kampuš N.J., Krajcar D., Markun B., Kočevar K. (2015). Determining the Polymer Threshold Amount for Achieving Robust Drug Release from HPMC and HPC Matrix Tablets Containing a High-Dose BCS Class I Model Drug: In Vitro and In Vivo Studies. AAPS PharmSciTech.

[47] Palanisamy M., Khanam J. (2011). Solid dispersion of prednisolone: Solid state characterization and improvement of dissolution profile. Drug Dev. Ind. Pharm..

[48] Nguyen M.N.U., Van Vo T., Tran P.H.L., Tran T.T.D. (2017). Zein-based solid dispersion for potential application in targeted delivery. J. Pharm. Investig..

[49] Reddy K.S., Prabhakar M.N., Rao K.M., Suhasini D.M., Subha M.C.S., Rao K.C. (2013). Development and Characterization of Hydroxy Propyl Cellulose/Poly(vinyl alcohol) Blends and Their Physico-Chemical Studies. Indian J. Adv. Chem. Sci..

[50] Eguchi N., Kawabata K., Goto H. (2017). Electrochemical Polymerization of 4,4-Dimethyl-2,2’-Bithiophene in Concentrated Polymer Liquid Crystal Solution. J. Mater. Sci. Chem. Eng..

[51] da Silva Peixoto T., Yamashita F., Bilck A.P., Carvalho G.M., Grossmann M.V.E. (2019). Crosslinking starch/oat hull mixtures for use in composites with PLA. Polimeros.

[52] Rials T.G., Glasser W.G. (1988). Thermal and dynamic mechanical properties of hydroxypropyl cellulose films. J. Appl. Polym. Sci..

[53] Veiga M.D., Cadorniga R., Lozana R. (1985). Thermal Study of Prednisolone Polymorphs. Thermochim. Acta.

